# Validation of vulnerability markers of dysfunctions in the socioemotional development of infants

**DOI:** 10.1590/1518-8345.2736.3087

**Published:** 2018-11-29

**Authors:** Daniel Ignacio da Silva, Débora Falleiros de Mello, Renata Ferreira Takahashi, Cody Stonewall Hollist, Verônica de Azevedo Mazza, Maria de La Ó Ramallo Veríssimo

**Affiliations:** 1 Universidade de Santo Amaro, São Paulo, SP, Brazil.; 2 Universidade de São Paulo, Escola de Enfermagem de Ribeirão Preto, PAHO/WHO Collaborating Centre for Nursing Research Development, Ribeirão Preto, SP, Brazil.; 3 Universidade de São Paulo, Escola de Enfermagem, São Paulo, SP, Brazil.; 4 University of Nebraska-Lincoln, College of Education and Human Sciences, Lincoln, NE, United States of America.; 5 Universidade Federal do Paraná, Departamento de Enfermagem , Curitiba, PR, Brazil.

**Keywords:** Health Vulnerability, Infant, Child Development, Developmental Disabilities, Development Disorders, Pervasive, Pediatric Nursing

## Abstract

**Objectives:**

to validate the vulnerability markers of dysfunctions in the socioemotional development of infants.

**Methods:**

study with a sequential exploratory mixed-method design. The vulnerability markers elaborated in the qualitative phase were analyzed by experts in the quantitative phase using the Delphi technique with a minimum consensus of 70%. Seventeen judges answered the questionnaire in the first round of analysis and 11 answered in the second round.

**Results:**

in the first round, two markers did not reach minimum consensus: the presence of instability in family relationships (66%) and delinquency and/or drug abuse by parents/caregivers (65%). In the second round, all markers were validated, with more than 90% agreement in most of the attributes, and reached the minimum consensus of 73%.

**Conclusion:**

the eight vulnerability markers reached the minimum consensus for validation, and a relevant instrument for infant care can be developed after assessing the reliability and clinically validating these markers.

## Introduction

The objective of this study was to validate the vulnerability markers of dysfunctions in the socioemotional development of infants. We attempted to construct an instrument that assessed dysfunctions in socioemotional development, which is determined by the maintenance or changes in social and emotional characteristics of children^1^ and characterized by the expression of emotions in social contexts, in the social triggers of emotional expressions, and in the social construction of emotional experience and understanding^2^.

Socioemotional development is related to the development of the brain and the interactions or proximal processes experienced by the child from birth^1^ and can be analyzed by evaluating developmental milestones from several domains, including attachment, social competence, emotional competence, and self-perception^3^.

The bioecological model of human development indicates that a child living in adverse conditions and in a disorganized environment is susceptible to developmental dysfunctions, including “recurrent difficulties in maintaining emotional control and integrating behavior in different developmental situations and domains”^1^. Therefore, child development is affected by biological and contextual factors^4^ ^-^ ^5^.

Developmental dysfunctions include a group of diseases characterized by intellectual, physical, and social-emotional problems^6^. These dysfunctions are related to brain disorders caused by genetic changes or lesions in the central nervous system, exposure to teratogenic agents, trauma, infections, severe nutritional deficiency, and neonatal hypoxia or ischemia^6^. Studies have confirmed that sociocultural, socioeconomic, psychosocial, and biological factors affect child development in all its dimensions, including socioemotional^4^ ^,^ ^7^.

The technologies available to monitor child development include scales based on markers and expected behaviors for different age groups. These technologies assess the child’s abilities but do not consider the factors that affect child development, leaving a significant gap in the analysis of dangerous situations.

The complexity of socioemotional development involves the concept of vulnerability, which is a set of conditions that make the child more susceptible to developmental dysfunctions due to the effect of individual, social, and programmatic dimensions^8^. The concept of vulnerability demands the proposition of interventions based on health needs, development of social responses, autonomy in care, preservation of health, and integrality and equity of health actions^9^.

The need to instrumentalize health professionals to identify vulnerabilities in child development led to the proposition of the following question: How can professionals assess the vulnerability to dysfunctions in the socioemotional development of infants?

The construction of markers may help health professionals apply the concept of vulnerability as an indicator of qualitative aspects of the health-disease process at the individual and community levels, and these markers allow proposing interventions that address social responses to dysfunctions^9^ ^-^ ^10^. The term “vulnerability marker” includes the interaction of subjective and contextual attributes in the health-disease process as social and historical phenomena^11^.

This study assumes that the use of markers as health technologies, based on vulnerability elements, can improve care and socioemotional development by strengthening proximal processes, which are the specific forms of interaction between children and their environment^1^.

The identification of these elements and characterization of the conditions of child development beyond the short-term performance, expressed in behaviors or developmental milestones, requires the inclusion and organization of these elements in an instrument applicable to the care practice. Therefore, the objective of this study was to validate markers of vulnerability to dysfunctions in the socioemotional development of infants.

## Method

This mixed-method study combined qualitative and quantitative methods^12^. A sequential exploratory design was used, including a first (qualitative) phase for marker construction and a second (quantitative) phase for content validation.

Vulnerability markers were elaborated in the qualitative phase. These markers are thematic categories of exposure factors that affect the socioemotional development of infants^13^ and are theoretically based on the context dimensions of the bioecological model of human development-microsystem, mesosystem, exosystem, and macrosystem^1^-and the Child Vulnerability Matrix for situations that jeopardize child development in the individual, social, and programmatic dimensions^8^. In this study, infants are children younger than two years.

Each marker is composed of a title, components, and an operational manual, and the function of the latter is to guide the application of the analytical instrument. The manual contains the definition of the markers, vulnerabilities, sources of information on the marker, and the criteria for defining the presence of the marker^11^.

**Figure 1:**
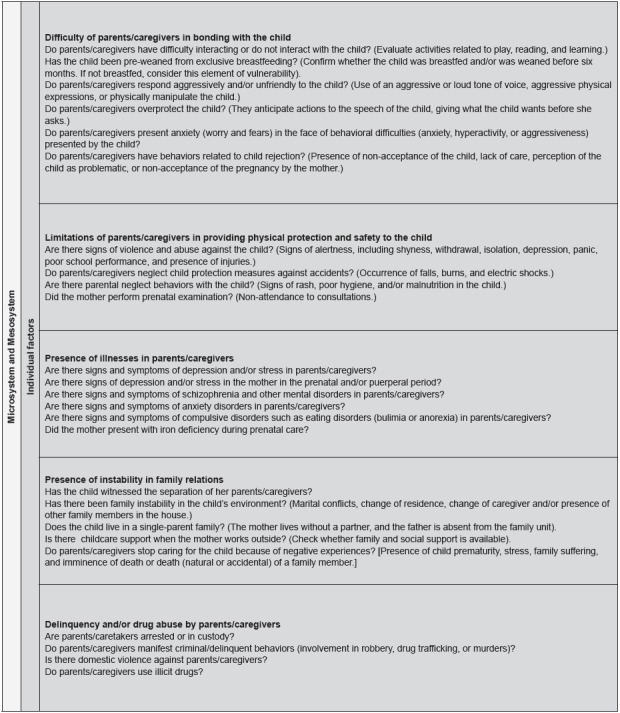
Original version of the vulnerability markers and their components related to the bioecology of development and individual vulnerability. São Paulo, Brazil, 2016

**Figure 2:**
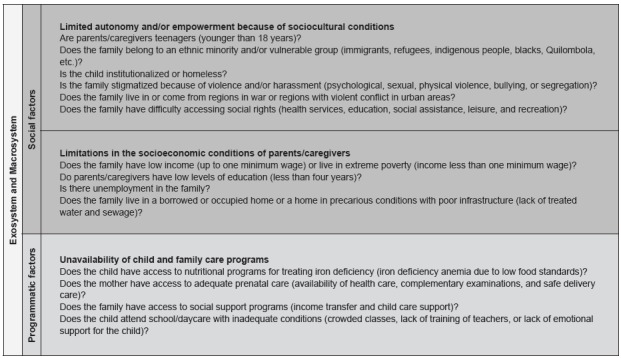
Original version of the vulnerability markers and their components related to the bioecology of development and social and programmatic vulnerability. São Paulo, Brazil, 2016

The original version of the vulnerability markers was sent to the experts for content validation. The markers are shown in Figures 1 and 2.

The Delphi technique^14^ was applied in the quantitative phase to validate the content of the markers, components, and operational manuals by researchers identified in the Platform Lattes who were specialists in socioemotional development. The selection criteria of the judges were the time of clinical experience and/or research on infant health, completion of undergraduate studies with a minimum duration of 5 years, and graduate studies in infant health.

Eighty-four nursing researchers and other health professionals were invited to assess the instrument because the concept of vulnerability is multidisciplinary. The invitations were made by sending an e-mail containing the Informed Consent Form (ICF) and the validation script of the markers in electronic format. Participants were considered the professionals who returned the ICF and completed the questionnaire within the deadline established for the first round. Two rounds of evaluation were necessary to reach the minimum consensus.

The questionnaire was developed using Microsoft Excel. The first page contained the ICF and guidelines for completing the questionnaire. All the content related to the markers was described in a spreadsheet, allowing participants to answer the questions using all available information.

The criteria used during validation to evaluate the attributes and relevance of the markers were simplicity, clarity, pertinence, and precision. The questions asked were 1. “Is the marker easily explained and understood?”, 2. “Can data on the marker be easily obtained?”, 3. “Does the marker effectively identify vulnerabilities to dysfunctions in the socioemotional development of infants?”, 4. “Can the marker be used in care practice?”, 5. “How important is this marker to identify infant vulnerabilities?“

The following questions were formulated to evaluate the attributes of marker components: 1. “Does the component adequately express the presence of a vulnerability in infants?”, 2. “Is the component constructed with simple and unambiguous expressions?”, 3. “Does the component differ from other components?”

The following questions were formulated to evaluate the operational manual: 1. “Was the marker and what it measures adequately described?”, 2. “This marker reflects vulnerabilities in individual, social, or programmatic factors. Do you agree with this statement?”, 3. “Are the sources of information accessible and adequate to obtain the data?”, 4. “Are the criteria adequately described and allow the same interpretation among the different health professionals who used the instrument?”

Only the “yes/agree” question was considered, excluding from the analysis the answers “yes, but requires revision/partial agreement” and “no/disagree.” The revisions necessary between each collection stage were made according to the suggestions of the judges.

Possible answers were agreement, partial agreement, or disagreement, and there was room for comments. Descriptive statistics were used for data analysis, and the minimum consensus was 70%^15^ ^-^ ^16^. The consensus is the expected result of the Delphi technique. Therefore, the definition of consensus criteria and the description of the degree of agreement and the validation results are essential^15^ ^-^ ^16^.

This study was approved by the Research Ethics Committee of the School of Nursing of the University of São Paulo via the Certificate for Ethics Assessment (Certificado de Apresentação para Apreciação Ética-CAAE) No. 57933816.8.0000.5392. The study complied with human research guidelines.

## Results

The first round of content validation was completed by 17 participants. Of these, 11 were nurses, two were physical therapists, two were occupational therapists, and two were psychologists. Most participants had a time of academic education longer than 10 years, with an M.S. and/or Ph.D. degree and experience in teaching, research, and care practice.

The judges returned the materials within 30 days and completed 95% of the questionnaires in the first round. The results of the assessments were tabulated according to pre-established parameters. The level of consensus of the judges in the first round is presented in Table 1.

**Table 1:** Minimum level of consensus of the judges in the first round of content validation. São Paulo, Brazil, 2017

Marker	Minimum level of consensus (%)		
	Operating manual	Attributes and marker relevance	Attributes of marker components
Difficulty of parents/caregivers in bonding with the child	93.0	75.0	73.0
Limitation of parents/caregivers in providing physical protection and safety to the child	94.0	81.0	75.0
Presence of illnesses in parents/caregivers	88.0	73.0	70.0
Presence of instability in family relations	94.0	87.0	66.0
Delinquency and/or drug abuse by parents/caregivers	81.0	64.0	65.0
Limited autonomy and/or empowerment because of sociocultural conditions	81.0	80.0	75.0
Poor socioeconomic conditions of parents/caregivers	93.0	94.0	76.0
Unavailability of child and family care programs	87.0	87.0	75.0

In the first round, the level of consensus of most of the assessed items was medium to high (70-94%). In addition to the objective answers, the judges provided 206 written suggestions, which were used in content review in the second round. The judges’ suggestions were related to the writing, presentation, and exemplification of the components.

The fourth marker component, “difficulty of parents/caregivers in bonding with the child,” was modified according to the judges’ recommendation: *The term “parental anxiety” does not seem to be the most appropriate*. *My interpretation is that this term indicates the exaggerated concern, maladjustment, or emotional imbalance of the parents due to the behavior of the child.* (J10)

The second and third marker components, “limitations of parents/caregivers in providing physical protection and safety to the child,” were drafted differently without the term “neglect” considering the following recommendation: *I suggest replacing the term “neglect” with another construct, such as “do not take the necessary measures.” This marker is important because it is common for families not to identify the risk factors for accidents.* (J10) The fourth component was rewritten according to the judge’s suggestion: *I suggest replacing the term “adherence” with “undergoing prenatal examination and prenatal care.”* (J10)

The number of components of the marker “presence of illnesses in parents/caregivers.” was reduced from six to three considering the recommendation: *All questions except the last one were related to mental health. However, does altered physical health affect childcare? In addition, considering that all these symptoms are related to changes in mental health, it may seem confusing: can stress, depression, and schizophrenia affect care in different ways? If so, why are these symptoms separated?* (J15)

The simplicity and expression of the fourth marker component, “presence of instability in family relations,” reached a consensus of 64%, which is lower than the minimum consensus. The component was changed according to the following commentary: *I suggest the following change: “(...) negative experiences within the family.” (J1) Describe the term “negative experiences” better and remove the terms related to mental health problems because they have already been included in another marker.* (J15)

It was suggested to include support for mothers in this marker: *I suggest leaving this item as “there is no support for childcare” and exclude the sentence “for the mother who works outside” because I consider that support is necessary for all mothers, regardless of working outside.* (J13) Therefore, the term “social support” was added.

The relevance of the first, third, and fourth components of the marker “delinquency and/or abuse by parents/caregiver” reached a consensus of 64%. The simplicity and expression of these components reached a consensus of 65%. The judges made the following suggestion: *Fulfillment of sentence because of the practice of criminal offenses. The inconsistency is related to the verb in the two tenses (present and past).*(J15) *Does this item indicate that caregivers suffer from domestic violence or the male partner is violent with the female partner?*(J4) *Review “there is presence.” I suggest including the question “Do parents/caregivers make use of psychoactive or other drugs?”*(J9)

With respect to the marker “limited autonomy and/or empowerment because of sociocultural conditions,” the following suggestion was accepted: *Is the difficulty related to the parents or the child? Autonomy/empowerment is also a limitation. I suggest leaving only the term “autonomy”*(J15). The fifth component of this marker was modified according to the judges’ recommendation: *I suggest adding “gangs or organized crime” to a situation closer to the “Brazilian war conflicts.” (J1) I suggest excluding the term “war” because it is not the reality of Brazil, and perhaps include the term “urban violence.”*(J13)

The first marker component, “poor socioeconomic conditions of parents/caregivers,” was modified according to the judges’ recommendation: *The question is repetitive. I suggest including the question: “Does the family have an income lower than the minimum wage*”? (J11) *I suggest rewriting the sentence, perhaps expressing the item as per capita income because a family with three members living on a minimum wage is different from a family with ten people living on a minimum wage.*(J13) The fourth component was modified according to the suggestions of one judge: *Can the family live in a borrowed or occupied house under normal conditions? I think what matters is the precarious situation. I suggest eliminating the first part of the sentence and including the sentence “The family lives in a precarious house.”*(J15)

After the inclusions and adaptations in the first round, the instrument was subjected to the second round of the Delphi technique. Of the 17 judges who participated in the first round, 11 participated in the second round. Of these, eight were nurses, one was a physiotherapist, and two were occupational therapists. The majority had a time of academic education longer than 10 years, with an M.S. and/or Ph.D. degree and experience in teaching, research, and care practice.

In the second round, the judges returned the materials within 30 days and completed 99% of the questionnaires. The level of consensus of the judges is presented in Table 2.

**Table 2:** Minimum level of consensus of the judges in the second round of content validation. São Paulo, Brazil, 2017

Marker	Minimum level of consensus (%)		
	Operating manual	Attributes and marker relevance	Attributes of marker components
Difficulty of parents/caregivers in bonding with the child	91.0	91.0	73.0
Limitation of parents/caregivers in providing physical protection and safety to the child	91.0	100.0	91.0
Presence of illnesses in parents/caregivers	100.0	91.0	91.0
Instability in family relations and poor social support	100.0	100.0	73.0
Violence and/or drug abuse by parents/caregivers	91.0	100.0	91.0
Limited autonomy of parents/caregivers because of sociocultural conditions	100.0	100.0	91.0
Poor socioeconomic conditions of parents/caregivers	100.0	100.0	82.0
Unavailability of child and family care programs	91.0	100.0	82.0

The level of consensus of most of the elements evaluated in the second round was high (82-100%), and two markers obtained the minimum consensus of 73%, which was higher than the established minimum, and the validation process was complete. In the last round, the judges sent 45 comments with suggestions on the writing of the components, and these suggestions improved the clarity and understanding of the instrument.

**Figure 3:**
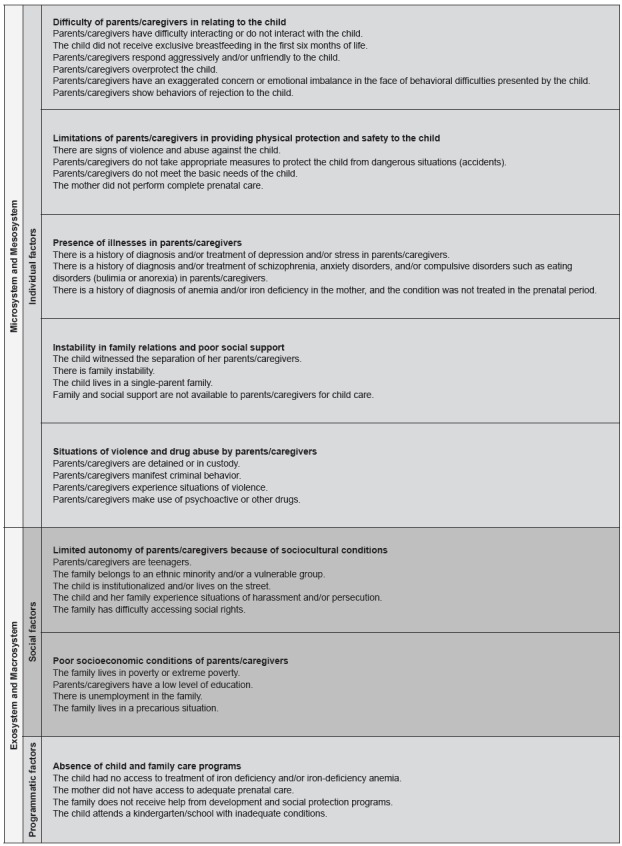
Final version of vulnerability markers and their components after content validation. São Paulo, Brazil, 2017

The markers of vulnerability to dysfunctions in the socioemotional development of infants and marker components of the final version are described in Figure 3. These elements were classified into three categories according to the contexts of the bioecological model of human development and vulnerability dimensions: individual (green), social (orange), and programmatic (blue).

## Discussion

The vulnerability markers were subjected to the Delphi technique and assessed by qualified professionals (with an M.S. and/or Ph.D. degree) with more than 10 years of academic training. These judges performed a critical analysis of the material and provided many suggestions (206 in the first round and 45 in the second). The questionnaire adherence rate was high (95% in the first round and 99% in the second round). These results corroborate the Delphi technique, whose application demands the recruitment of experienced, socially critical, and professionally self-critical judges who can make significant changes and adaptations to the analyzed material^17^ ^-^ ^18^.

The number of participants in the first and second rounds was considered pertinent by the literature, which defines a minimum of 10-15 specialists to obtain a set of high-quality opinions^18^. Therefore, the markers were appraised by a diverse group of judges from different areas of practice, allowing a thorough analysis of the material.

Although this instrument was initially intended for use in the area of nursing in infant health, the evaluation and improvement of the quality of these parameters by psychologists, occupational therapists, and physical therapists were relevant considering that psychosocial development is multidisciplinary. This multiprofessional evaluation is recommended by the Delphi technique, which makes these parameters accessible to a diverse and geographically dispersed population, allowing the provision of different opinions^19^.

Failure to reach the expected consensus in the first round for all analyzed items may be justified by the high number of comments from the judges because many sentences were written using terms deemed inappropriate. The achievement of a minimum consensus of 73% and the comparatively lower number of comments in the second round demonstrated that the material was more appropriate.

With regard to changes in the content of the marker components “difficulty of parents/caregivers in bonding with the child” and “limitations of parents/caregivers to provide physical protection and safety to the child,” the modifications allowed a better understanding of the limitations of childcare. These limitations affect the type and quality of care and the interactions between parents and infants^1^ ^,^ ^20^.

With respect to the marker “illnesses in parents/caregivers,” the judge’s recommendation to include the mental health conditions to facilitate their identification by professionals was considered adequate. The presence of mental disorders is related to the lower degree of affection for the infant and the development of weak bonding^20^.

With respect to the marker “presence of instability in family relations,” which did not reach the minimum consensus, the judges’ suggestions were pertinent because negative experiences might lead to vulnerabilities in caregivers, limit childcare support, and lead to neglect and exposure of the child to dangerous situations^21^ ^-^ ^22^.

With regard to the marker “situations of delinquency and/or drug abuse by parents/caregivers,” which also did not reach minimum consensus, addressing the drug abuse of parents/caregivers is relevant to identify situations that are adverse to the socioemotional development of the infant^23^ ^-^ ^24^. Similarly, home violence suffered by caregivers may impair childcare and consequently the bonding with the child ^(^ ^25^. Therefore, the proposed modifications avoid erroneous interpretations of professionals when using this instrument.

With regard to the marker “limited autonomy of parents/caregivers because of sociocultural conditions,” emphasizing the autonomy of caregivers in the title of the marker is relevant because this marker reflects the caregivers’ ability to care for the child^8^ ^,^ ^22^ ^-^ ^23^. Adaptations were made in the component of this marker to characterize violence as a set of conditions that imposed stigma and oppression on caregivers^23^.

The changes in the marker “poor socioeconomic conditions of parents/caregivers” are pertinent because professionals should understand that growth under conditions of poverty exposes the child to poor living conditions. Therefore, the socioeconomic status of the family directly affects childcare^4^ ^,^ ^8^.

The high agreement rates for vulnerability markers starting in the first round of analysis indicate that such markers are comprehensive for the bioecology of development^1^ and vulnerability^8^.

The reliability and clinical validation of the vulnerability markers presented in this study need to be assessed beyond the consensus of expert opinions, and this validation will increase the applicability of primary health care practices to promote the socioemotional development of infants^8^.

## Conclusion

The markers of vulnerability to dysfunctions in the socioemotional development of infants was validated after two rounds of the Delphi technique, and most markers, components, and operational manuals reached a high rate of agreement (>90%) and a minimum level of consensus of 73%.

The consensus reached using the Delphi technique allows testing this technology in clinical practice to assess its reliability by professionals to create care models based on the actual health needs of infants and minimize exposure factors and the vulnerability to dysfunctions in socioemotional development.

One of the limitations of this study was that the markers were based on scientific evidence that might not account for the totality of current vulnerability situations; therefore, the reliability of these markers needs to be evaluated. Longitudinal studies that allow the routine clinical validation of vulnerability markers by health professionals during child and family care are necessary.

For nursing practice, the application of this instrument allows constructing a scale of vulnerability, identify new diagnoses in nursing, and elaborate intervention plans that promote the socioemotional development of infants by nurses and other professionals.
